# The Book World of Medicine and Science

**Published:** 1905-09-23

**Authors:** 


### The Book World of Medicine and Science.

A Manual of Clinical Chemistry, Microscopy, and Bacteriology. By Dr. M. Klopstock and Dr. A. Kowarsky, of Berlin. Translated by Thew Wright, M.D. (Rebman, Limited. Price 8s. net.)

In the preface to this book the authors state that they desire to place a concise manual dealing with these subjects in the hands of the practitioner and student with no intention of replacing larger text-books: hence we find that the subject-matter of the book is condensed, reading in this respect like notes, but at the same time no important details are omitted. The book quite satisfies a want that must be felt by many for a short text-book dealing with both bacteriology and clinical chemistry, and microscopy. It should prove very useful to those reading for the higher degrees in medicine, such as the final M.B. of the London University, in which a special examination is held in the subjects here dealt with. Being written by German professors it might be expected that some of the methods described would be quite new to the English student, but nearly all the important ones are the same as those employed in English laboratories: exceptions are to be found in the estimation of sugar and urea in the urine, the estimation of sugar by means of the polarimeter and by fermentation is given, but no mention is made of the use of Fehling's solution, either with a burette or the simpler and quicker apparatus invented by Carwardine. In the estimation of urea Jolles' apparatus is used, which seems to be much more complicated than that which bears Dupré's name, though it is on the same principle. The authors take each secretion of the body separately and devote a whole chapter to it, in which the chemistry, microscopical appearance, and bacteriology of the secretion are thoroughly discussed. In addition, a chapter is given to the bacteriological examination of the skin in disease and another to various bacteriological methods, *e.g.* the preparation of stains, culture media, etc. We notice in the chapter on the examination of the fæces that through some error, the lengths of the various tape-worms that are found in man are very much understated, but this is probably a clerical mistake. We can thoroughly recommend this book to both student and practitioner, and we are sure it will be found a most useful laboratory guide.

International Clinics. Vol. I. Fifteenth Series. 1905. Edited by A. O. J. Kelly, M.D. (Philadelphia and London: J. B. Lippincott Company. 1905. Pp. 312. Price 10s. 6d.)

As number succeeds number, and a high level of excellence is maintained by all, it becomes scarcely necessary to do more than call attention to each new volume of these clinics when it is published. There is little room in the representative of the series which is now before us for adverse criticism, while there is much that is worthy of commendation. A noteworthy feature is the article dealing with the progress of medicine and surgery during 1904. This retrospect has been most judiciously compiled, and without any surplusage shows in as short a space as possible the entire trend of medical discovery and research during that year. The remainder of the volume is occupied by contributions from authors living in America, the Continent, and the United Kingdom. We have not space to mention each article specifically, and where all is good it would be invidious to choose individual papers for special mention.

Laryngeal Phthisis. By Lake and Barwell. (London: Baillière, Tindall and Cox. 1905. Second Edition. Pp. 120. Illustrated. Price 6s. 6d.)

This is a well-written and thoroughly up-to-date treatise. Tuberculosis of the larynx is fully discussed, especially as regards its treatment, to which thirty-four pages are given. The importance of an early diagnosis is demonstrated, and great stress is laid on the good results to be obtained by early operative treatment. The book is well illustrated and ought not to fail in the author's object "to induce the profession to give more attention to this painful disease."

Standard Chemical Disinfectants. By Wolf Defries.

This brochure represents yet one more plea for the standardisation of disinfectants according to their bactericidal power. Although issued in the interests of a well-known firm of manufacturers, the arguments which it puts forward are none the less cogent. Apart from the fact that some of the substances extensively advertised under the name of disinfectants, and largely used as such by the public, have little or no disinfectant power at all, it would be a great gain to the user, whether a medical man or not, to know the precise antiseptic value of the various reagents at his disposal for the destruction of pathogenic organisms. Until this knowledge is available it will be impossible to be sure of combining efficient disinfection with a minimum expenditure. Indeed, it is strange that in spite of all the modern achievements of bacteriology the employment of reagents for destroying micro-organisms should be so haphazard and so absurdly unscientific as it is at the present day.

Natural Science in Hygiene. By James Rodger Watson, B.Sc., M.D., D.P.H. (Bristol: John Wright and Co. 1905. Pp. 62. Price 1s. 6d. net.)

The object of the author is to place before public health students the life histories of those members of the vegetable and animal kingdom which are capable of infecting man. The descriptions of the various parasites are short, clear, and to the point. Candidates for the D.P.H. wishing to revise this part of their work will find Dr. Watson's little book exceedingly useful.

The Water Supply of Villages and Small Towns. By H. C. H. Shenton, M.S.E. ("Local Government Journal" Office. Pp. 52. Price 6d. net.)

The problem of supplying pure water to villages and small towns is often both a serious and a trying one. Mr. Shenton has studied the question from all points of view, with the result that he has written this book. To use his own words, "it is a collection of practical notes, and does not treat with the whole subject of water supply." As such, surveyors and others having to deal with small water supplies will find this book of service to them in their work. A full index is provided.

### BOOKS RECEIVED.

J. B. Lippincott Co.

